# Defective Fas Expression on Bone Marrow Derived Cells Alters Atherosclerotic Plaque Morphology in Hyperlipidemic Mice

**DOI:** 10.15190/d.2015.29

**Published:** 2015-03-31

**Authors:** Nobuhiko Kubo, Sara McCurdy, William A. Boisvert

**Affiliations:** Department of Clinical Laboratory Medicine, Omiya Medical Center, Jichi Medical School, Japan; Center for Cardiovascular Research, John A. Burns School of Medicine, University of Hawaii, Honolulu, Hawaii, USA

**Keywords:** Fas, FasL, inflammation, macrophages, atherosclerotic plaque

## Abstract

Fas (CD95) is a member of the TNF-receptor family expressed on a wide range of cells. Interaction of Fas with its receptor, Fas ligand (Fas-L), stimulates an intracellular cascade of events that leads to apoptosis. Because apoptosis of inflammatory cells plays a key role in atherosclerosis we sought to determine the role of Fas in the development of atherosclerosis by repopulating the bone marrow cells of atherosclerosis-prone low density lipoprotein receptor null (LDL-R-/-) mice with either cells from lpr mice (lpr-BMT) that have defective Fas expression or from control mice (WT-BMT). The lpr-BMT mice exhibited no peripheral blood Fas expression 4 weeks after BMT. After consuming an atherogenic diet for 16 weeks, lpr-BMT mice developed atherosclerotic lesions characterized by smaller fibrous area with thinner fibrous cap and less TUNEL-positive staining compared to WT-BMT mice, although overall lesion size in lpr-BMT mice was similar to that of WT-BMT mice. Examination of a series of human atherosclerotic lesions revealed that many Fas-positive cells were colocalized with CD68-positive macrophages. Although apoptotic cells were rarely observed in the foam cell-rich fatty streak lesions, apoptotic CD68-positive macrophages in advanced lesions were detected in areas rich with inflammatory cells near the necrotic core. These observations suggest that Fas expression by the macrophages in atherosclerotic lesions can influence the plaque morphology towards a more fibrous type.

## INTRODUCTION

Fas (CD95), a type I membrane protein, is a member of the TNF-receptor family that is widely expressed in a variety of cells^1,2^ . Crosslinking of Fas on the cell by interaction with its receptor, Fas ligand (Fas-L), stimulates an intracellular cascade of events that leads ultimately to apoptosis, the programmed cell death events that serve to maintain tissue homeostasis in the evolving paradigm^3,4^. Activation of Fas can also result in non-apoptotic responses such as cell proliferation or activation^5-7^. In atherosclerotic lesions, Fas is expressed on smooth muscle cells (SMC), macrophages and endothelial cells^8-10^.

The lpr mice were identified as a mutant strain of MRL mice that showed abnormal lymphoproliferation (lpr) due to a defect in lymphocyte apoptosis. These MRL-Fas^lpr^ mice were found to have defective Fas expression caused by a retroposon insertion in the Fas gene that leads to a defect in negative selection of self reactive T cells^11,12^. This defect, which is expressed in all cells including macrophages, contributes to the development of autoimmune disease in MRL mice^13^. Several studies have utilized these MRL/lpr mice to demonstrate the involvement of chronic inflammation by autoimmunity in atherogenesis^14,15^. MRL/lpr mice fed a cholesterol-containing high fat diet become hyperlipidemic and have a higher incidence of atherosclerotic lesions of renal and aortic branch arteries^16^. MRL/lpr mice also display an exacerbated coronary artery disease when given the atherogenic diet compared with normal mice^14^. These studies suggest a proatherogenic role of chronic inflammation and of immune cells such as T cells, B cells and natural killer cells^17,18^.

Lipid rich environments such as the atherosclerotic lesion that result from hyperlipidemia can affect macrophage viability. Lipoproteins can have differing influences on the fate of macrophages depending on the choice of lipid reagents or modification process of lipoproteins. For example, free cholesterol loading causes macrophage death by apoptosis through the Fas pathway^19^. Oxidized lipoproteins can also cause macrophage cell death^20,21^. Apoptotic cells are localized in necrotic cores that contain lipid-loaded macrophages as well as modified lipoproteins^22^. Interestingly, our previous studies have shown that macrophage apoptosis is suppressed by both native and modified lipoproteins^23^. This indicates that macrophage apoptosis is a complex process and that its trigger in atheromatous lesions is incompletely understood.

Several studies in humans have identified morphological features that characterize the unstable atheroma, including a thin, acellular, eccentric fibrous cap and large necrotic core of lipid and cellular debris^24,25^. These studies indicate that the cellularity can potentially affect the structure and stability of atherosclerotic lesions. Accordingly, the regulation of cell activation, survival and death through Fas pathway in macrophages may impact atherosclerosis by affecting the stability of the neointima. To examine how macrophage apoptosis may affect the atherosclerotic development, we utilized mice in which apoptosis is compromised by the lack of Fas. We used a chimeric approach in which atherosclerosis-prone LDL-R-/- mice^26,27^ were subjected to bone marrow transplantation with MRL/lpr marrow cells to study the role of leukocyte-specific Fas expression in atherosclerosis. In contrast to control LDL-R-/- mice that received wild type bone marrow cells (WT-BMT mice), the LDL-R-/- mice that received lpr bone marrow cells (lpr-BMT mice) displayed atheromatous lesions that contained a much more prominent acellular neointima and a thinner fibrous cap area. We also explored the expression of Fas in human atherosclerotic tissue samples and show that Fas is expressed abundantly. Immunohistochemical staining of serial sections revealed that Fas expression is particularly robust in the macrophages within the lesion.

## MATERIALS AND METHODS

### Animals and facilities

Wild type mice and lpr mice both on the C57BL/6 background were obtained from Jackson Laboratories (Bar Harbor, ME). LDL-R-/- mice also on the C57BL/6 background were initially purchased from Jackson Laboratories (Bar Harbor, ME) and bred in house. All mice were housed 4 per cage in a specific pathogen free room. They were kept on a 12 hour light-dark cycle, and were fed ad libitum either a chow diet (diet #5015, Harlan Teklad, Madison, WI) or an atherogenic high fat diet (HFD) that contained 15.8% (wt/wt) fat, 1.25% (wt/wt) cholesterol and no cholate (diet #94059; Harlan Teklad). The animals were bled under Metofane-induced anesthesia after an 8 hour fast by retro-orbital puncture into heparin coated capillary tubes. The blood was centrifuged at 3,000 x g for 5 min at 4°C and the separated plasma was stored at -20°C. The cells were used immediately for flow cytometry as described in the next section. All procedures were in accordance with institutional guidelines.

### Flow cytometry

Whole blood (0.1 ml) containing approximately 1 x 10^6^ peripheral blood leukocytes was washed in PBS containing 2 % fetal bovine serum (FBS-PBS) and the cell suspension incubated for 30 min at 4°C with Fact receptor blocking solution (Fc block: CD16/32, clone 2.4G2, PharMingen) and with biotin-labeled monoclonal anti-Fas antibody (clone Jo2, PharMingen) or biotin-labeled IgG (anti-trinitrophenol (TNP) as irreverent control; clone G235-2356, PharMingen) in 70 ml of FBS-PBS. After the cells were washed with FBS-PBS they were incubated with Streptavidin-PE (Vector) for 30 minutes at 4°C, and the red blood cells lysed by exposure to 0.14 M ammonium chloride. Cell- associated fluorescence was analyzed with a FACS system (Becton Dickinson) using CellQuest software (Becton Dickinson).**

### Bone marrow transplantation

Bone marrow transplantation (BMT) was performed as described previously^26,27^. Briefly, 6-wk-old male LDL-R-/- mice^28^ were subjected to 1,000 rad of total body irradiation to eliminate their bone marrow-derived cells. Donor marrow cells used for repopulation of the irradiated mice were isolated from lpr and wild type mice. The recipient LDL-R-/- mice that were injected I.V. with 2 x 10^6^ bone marrow cells from lpr and wild type donors were designated lpr-BMT mice and WT-BMT mice, respectively. After BMT, all mice were fed a chow diet. Four weeks later they were bled and fed the atherogenic high fat diet. Animals were sacrificed after they had consumed the atherogenic diet for 4, 11 and 16 weeks.**

### Assessment of atherosclerosis in aortic valves and aortas

Methods used to quantify atherosclerosis in the aortic valves are detailed elsewhere^29^. In brief, aortas from the proximal end to the renal artery were dissected out, cleaned of fascia and stored at -80°C. OCT-embedded hearts were sectioned in a cryostat until all three leaflets of aortic valve were visible within the aortic valve. From this point, 10 mm sections for approximately the next 300 mm of the valve region were collected on Superfrost slides (Fisher Scientific). The lipid-rich lesions were visualized by staining the sections with oil red O followed by a hematoxylin counter-stain. A total of five sections taken every 40 mm were used to quantitate the lesion area using a computer-assisted video imaging system. The mean aortic valve lesion area of each mouse was calculated as the average area of the five heart sections. General morphology of the sections was examined by staining with trichrome and quantifying the areas stained using video imaging system.

Detailed morphological examination of atherosclerotic tissue sections of each group of mice revealed that differences in morphology were limited only to the more advanced stage of the disease. Thus, eight such representative aortic valve sections containing lesions that resembled human type IV lesions as classified by the AHA/ATVB’s committee of vascular lesion^30^ were used for the quantitation of lesions. Examples of these lesions are shown in Figure 4. The tissue sections were stained with H&E and reveals the necrotic core as white vacuous space in the neointima, whereas the cellular, fibrous areas are colored brightly.

Aortas from the proximal end to the renal artery were dissected out, cleaned of fascia and stored at -80°C for later evaluation of cholesterol content. Cholesterol content of the dissected aorta was assessed by thin layer chromatography as described previously^31^.

### Immunohistochemitry and TUNEL staining

Paraffin-embedded sections were used for morphology and TUNEL staining whereas frozen sections were used for quantification of fibrous area of aortic valve lesions. Serial sections were used to identify smooth muscle cells by immunostaining for α-actin. Immunohistochemistry was performed with antibodies specific for Fas [FAS (x-20)-G, goat polyclonal IgG, Santa Cruz Biotechnology]; smooth muscle cell (α-actin, clone 1A4, EPOS, HRP, Dako Corp.); and macrophage (MOMA-2, Serotec, Raleigh, NC). Biotinylated secondary antibody (biotinylated anti-Rat IgG, Vector) was used for MOMA-2. Fas signals were amplified using the Tyramide signal amplification (TSA) kit (NEN, Beverly, MA) according to manufacturer’s instruction, and the color development was performed with use of ABC Elite kit and AEC kit (Vector).

TUNEL staining was performed with the In Situ Apoptosis Detection Kit (ApoTagR Plus Peroxidase in Situ Apoptosis Detection Kit, Intergen, Purchase, NY). Briefly, paraffin-embedded sections were deparaffinized and were treated for 15 min at room temperature with proteinase K (20 mg/ml). The sections were reacted with TdT enzyme (55 ml/5 mm^2^) at 37°C for 1 hr to tail the 3'OH termini of the DNA with digoxigenin-aNTP. Anti-digoxigenin, peroxidase conjugate was added and the complex was visualized by enzymatic color development. Positive staining was monitored by simultaneous staining of testis tissue.

### Immunohistochemical and morphological studies of human atherosclerotic tissues

Human atherosclerotic tissues were from 11 cases (8 cases with cervical artery stenosis by directional atherectomy of clinically narrowed cervical arteries and 3 cases with dissecting aneurysm by surgical resection). All of the tissue samples were from primary lesions that had not received previous intervention. The tissues were fixed in the 3 % formalin buffer immediately after collection. The paraffin sections were then dewaxed and rehydrated. Prior to reacting primary antibody, the sections were incubated with 5 % normal goat serum. Primary antibodies utilized included anti-CD68 (clone PG-M1, Dako Corp.) for the identification of monocytes/macrophages, alkaline phosphatase conjugated anti-α-smooth muscle (a-SM) actin (clone 1A4, Sigma Chemical Co.) for detection of smooth muscle cells (SMCs), and normal mouse IgG (Dako Corp.) as a control. The primary antibody for detection of Fas was a mouse monoclonal anti-human Fas antibody, clone APO-1 (Dako Corp.), diluted 1:500. Subsequent staining steps were performed by using the indirect avidin-biotin horseradish peroxidase or alkaline phosphatase visualization methods (LSAB2 kit, Dako Corp.). Sections to be stained for Fas were microwave treated for antigen retrieval before applying the primary antibodies, and the signals were amplified using the catalyzed signal amplification system (Dako Corp.). Apoptotic nuclei were identified by the single strand (ss)-DNA staining in which the sections were incubated with anti ss-DNA (rabbit polyclonal anti-ssDNA, Dako Corp.)^32^ 1:200 with 5% goat serum containing PBS for 1 hour at 37 °C in a humidifying chamber. After rinsing in PBS, the sections were incubated with biotinylated secondary antibodies, and exposed to biotin-POD. The sections were reacted with diaminobenzidine for color development. Results by ss-DNA stain and fragmented DNA in situ stain by TUNEL method were the same (data not shown). In non-atherosclerotic carotid arteries, immunohistochemical evaluation showed negligible or scattered staining of apoptotic marker of ss-DNA (data not shown). Morphological determinations of atherosclerotic lesions were determined according to histological classifications^30^.

### Plasma IgG, cholesterol assay and statistical analysis

Plasma total IgG levels were measured with the sandwich ELISA using polyclonal anti-mouse IgG (ImmunoPure^®^, Alkaline phosphatase labeled Goat anti-mouse IgG (H+L), Pierce). Enzymatic measurements of total cholesterol were performed on stored plasma with a kit (Sigma Chemical Co.) according to the manufactures directions. HDL cholesterol was measured by a precipitation method^31^. Statistical significance was determined using analysis of variance (ANOVA).

## RESULTS

### Repopulation of LDL-R-/- mice with donor bone marrow cells

**Figure 1:**
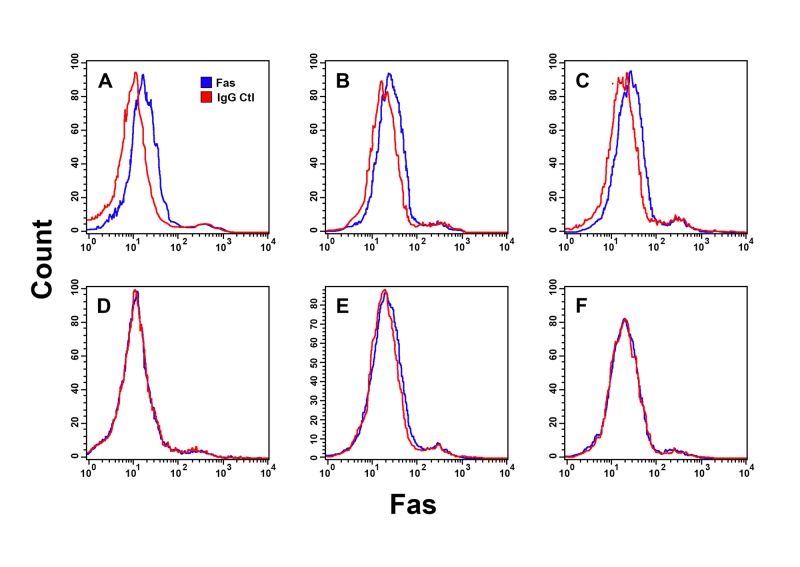
Fas expression in peripheral blood leukocytes of bone marrow recipient mice Representative results from one donor mouse as well as two mice from each group are shown as follows: wild type donor mouse (A), WT-BMT mice (B, C), lpr mouse (D), lpr-BMT mice (E, F). Red lines represent Fas staining whereas blue lines represent control samples with anti-TNP antibody.

**Figure 2:**
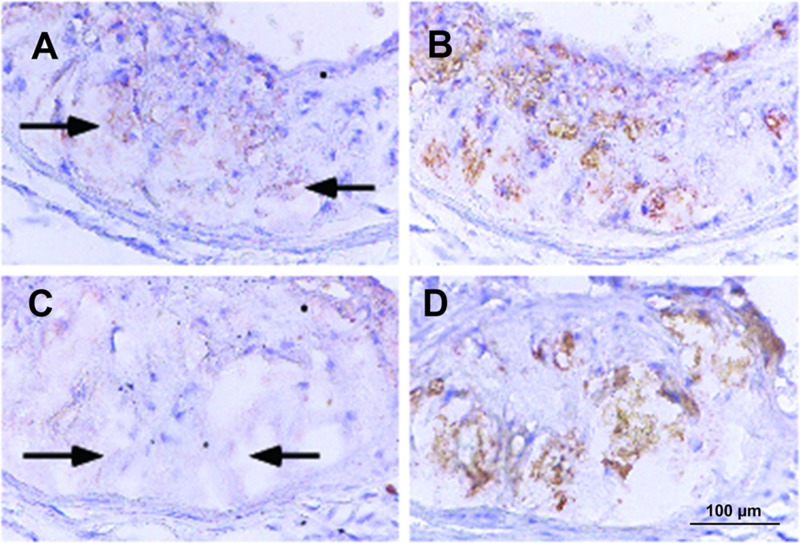
Fas expression in representative serial section of aortic valve lesions Tissues from WT-BMT mice (upper panels) and lpr-BMT mice (lower panels) are shown. Brown stain represents Fas (A, C) and MOMA-2 (B, D) (x100). Fas (arrows in A) was detected in the areas of MOMA-2-positive foam cells (B) of WT-BMT mice on adjacent tissue sections. However, Fas (arrows in C) was absent in the MOMA-2 positive areas of lpr-BMT mice (D). Fas was also detected in the areas negative for MOMA-2 (most likely smooth muscle cells) from both group of mice (A, C).

Flow cytometric analysis of Fas expression by peripheral blood leukocytes was used to confirm repopulation of the irradiated and bone marrow transplanted mice. Four weeks after BMT, leukocytes from WT-BMT mice were positive for Fas (**Figure 1** [B, C]) and this was similar to control LDL-R-/- mice that did not receive BMT (**Figure 1** [A]). In contrast leukocytes from lpr-BMT mice demonstrated only background staining for Fas (**Figure 1** [E, F]) and this was similar to staining seen in lpr mice (**Figure 1** [D]). Absence of Fas expression in lesion macrophages of lpr-BMT mice was confirmed by colocalization of Fas and MOMA-2 staining (**Figure 2**). Serial sections from WT-BMT lesions showed colocalization of Fas positive cells (arrows in **Figure 2** [A]) and MOMA-2 positive cells (**Figure 2** [B]). In contrast, Fas expression was largely negative in lpr-BMT lesions (arrows in **Figure 2** [C]) despite the presence of many MOMA-2 positive cells in the same region (**Figure 2**, [D]). These results not only confirmed successful repopulation in our chimeric mice but also showed that indeed Fas is not expressed in the lpr mice in accordance with previous reports showing defective expression of Fas in the lpr mice^12,33^. Although there may be weak Fas-positive staining in Moma-2-negative cells including smooth muscle cells within the lesions (as reported by others^8,9^), the most intense staining was associated with macrophages.

### Morphology of the atherosclerotic lesion

**Figure 3:**
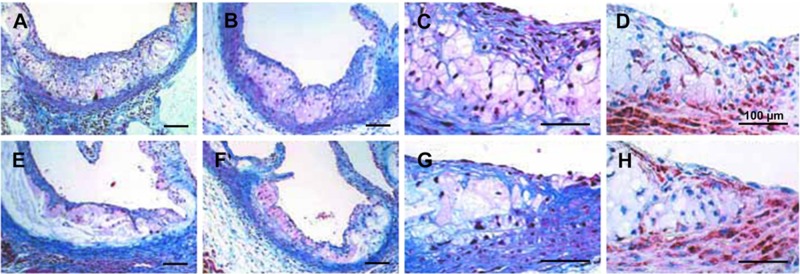
Trichrome staining of two representative valve lesions of WT-BMT mice (upper panels) and lpr-BMT mice (lower panels) Red stain represents a-actin (D and H). Magnifications of x100 (A, B, E, F) and x250 (C, D, G, H) are shown. Necrotic core areas from lpr-BMT tissues appear largely acellular with thin distinct borders (stained dark blue with trichrome stain) (E, F, G). The smooth muscle cell distribution was limited to a thin fibrous layer in the lesion from lpr-BMT mice (H) compared to a more even distribution of these cells in the tissues of WT-BMT mice (D).

Two representative valve lesions stained with trichrome stain are shown in **Figure 3**. There was a striking difference in the morphology of lesions between the two BMT groups of mice. Not only were the necrotic core areas in the atheroma of the lpr-BMT mice much larger (**Figure 3** [E, F]) compared to WT-BMT mice (**Figure 3** [A, B]), but the lesions from lpr-BMT mice were more acellular. Furthermore, 33% of lesions from WT-BMT mice that had consumed the atherogenic diet for 11 weeks consisted almost entirely of foam cells, whereas all of the lesions from lpr-BMT mice had only occasional foam cells. In these lesions the necrotic core areas were larger and the fibrous areas were thinner in the lpr-BMT mice. The layer of connective tissue between the necrotic core and the outer luminal space of the WT-BMT lesion was thick and cellular with well preserved extracellular matrix (stained light blue with trichrome stain) (**Figure 3** [A-C]). In contrast the necrotic core from lpr-BMT mice had many areas of disrupted connective tissue and thin border (stained dark blue with trichrome stain) between the necrotic core and the fibrous area (**Figure 3** [E-G]). While α-actin positive SMCs in the shoulder areas were generally distributed throughout the dense regions of WT-BMT lesions (**Figure 3** [D]) SMC (stained red) distribution was limited to the fibrous areas that formed a distinct but thin border with the necrotic core in the lesions from lpr-BMT mice (**Figure 3** [H]).

**Figure 4:**
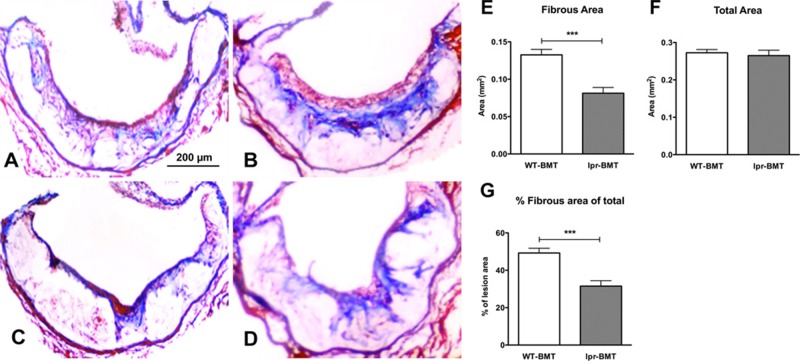
Representative examples of aortic sinus sections of murine type IV valvular atherosclerotic plaques from WT-BMT mice (A, B) and lpr-BMT mice (C, D) showing morphological differences and measurements Trichrome-stained blue and red areas bordering the lumen indicate fibrous area whereas the white, vacuous areas below them are designated as necrotic cores. Fibrous areas (FA) stained in blue as well as total areas (TA) of the lesion and the percentage of FA/TA of eight lesions from each group were calculated from these types of histological tissue samples and are quantified in graphs E-G. The percentage of the area to total area was calculated with use of NIH image analyzer, as described in the method section. FA, Fibrous area; TA, Total area. (*** p<0.001).

Quantification of fibrous area (FA; the blue area containing collagen fiber), total area (TA) and the percentage of FA/TA of eight lesions from each group is shown in **Figure 4**. Mean FA of the lpr-BMT lesions (0.08±0.02 mm^2^) was significantly smaller than that from WT-BMT lesions (0.13±0.02 mm^2^, p<0.001). While the TA was not different between the two groups, the mean FA/TA percentage of the lpr-BMT mice (31.5±8.2 %) was significantly smaller than that of WT-BMT mice (49.2±7.2 %, p<0.001).

### Cell death events in atheromatous lesions

**Figure 5:**
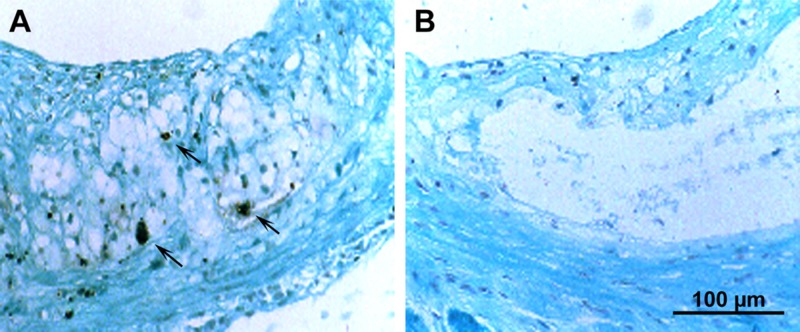
TUNEL staining of two representative atheromatous valve lesions of WT-BMT mice (upper panels) and lpr-BMT mice (lower panels) (x250) WT-BMT mice had TUNEL positive cells in the lipid rich areas of atheromatous lesions (A, indicated by arrows), whereas TUNEL positive cells were absent in atheromatous lesions collected at the same time from lpr-BMT mice (B).

TUNEL staining was performed to investigate apoptosis in the valve sections from mice that were fed the high fat diet for 4, 11 and 16 weeks. Consistent with reports claiming that apoptotic events are less frequent in earlier lesions compared to advanced atherosclerotic lesions^34-36^, the number of TUNEL positive cells in fatty steak lesions in WT-BMT mice fed the high fat diet for 4 weeks was considerably lower than the number found in lesions collected from animals fed the high fat diet for 11 or 16 weeks. Fatty streak lesions in both groups of mice fed the high fat diet for 4 weeks showed a similarly low frequency of TUNEL positive cells. After consuming the high fat diet for 11 weeks, however, WT-BMT mice had TUNEL positive cells in the lipid rich areas (**Figure 5** [A]), whereas TUNEL positive cells were absent in the similar areas collected at the same time from lpr-BMT mice (**Figure 5** [B]). Similar results were seen also in atherosclerotic lesions collected from animals fed the high fat diet for 16 weeks (data not shown).

### Growth, plasma lipid and atherosclerosis in lpr-BMT mice

All mice were kept in specific pathogen free conditions after BMT and exhibited similar growth patterns (**Table 1**). After consuming the high fat diet for 11 weeks, there was no difference in average body weights of WT-BMT mice (n=12) compared to lpr-BMT mice (n=10). Plasma cholesterol levels were similar in the two groups (**Table 1**). The assessment of aortic valve lesion areas in animals that consumed the high fat diet for 11 weeks revealed that the mean lesion areas of WT-BMT (0.51±0.11x10^6^/mm^2^) and lpr-BMT mice (0.52±0.14x10^6^/mm^2^) were similar. Cholesterol content by TLC analysis (**Table 1**), plasma total IgG (2.53±1.31 mg/ml of WT-BMT mice and 2.45±0.82 mg/ml of lpr-BMT mice, p=0.79) and HDL-cholesterol (90.1±47.2 mg/dl of WT-BMT mice, and 94.0±19.7 mg/dl of lpr-BMT mice, p=0.62) at 16 week high fat diet did not differ between two groups of mice.

**Table 1:** Growth, Plasma and Aorta Lipids of WT-BMT and lpr-BMT mice

Weeks on high fat diet	WT-BMT (n=12)	lpr-BMT (n=10)	P
Body weight (g)			
0	17.3 ± 1.2	17.9 ± 1.0	0.197
4	18.7 ± 1.3	19.3 ± 1.2	0.299
8	19.0 ± 1.4	18.4 ± 0.9	0.249
11	20.0 ± 1.6	19.2 ± 1.4	0.236
			
Total plasma cholesterol (mg/dL)			
0	264.0 ± 16.2	256.2 ± 23.3	0.361
4	1085.9 ± 175.0	973.4 ± 71.3	0.060
8	1042.8 ± 110.4	1108.9 ± 108.5	0.163
11	1052.4 ± 122.5	1196.7 ± 124.2	0.128
			
Aortic Lipid Contents (mg/aorta)			
Free Cholesterol	8.9 ± 1.8	17.8 ± 14.9	0.659
Cholesteryl Ester	40.1 ± 36.3	48.2 ± 16.3	0.530

### Lesion cell apoptosis related to plaque morphology in human atheroma

We examined a total of 11 human atherosclerosis samples (8 cases with cervical artery stenosis by directional atherectomy of clinically narrowed cervical arteries and 3 cases with dissecting aneurysm by surgical resection). Out of these, five showed lesions dominated by SMC proliferation and tight connective tissue with few macrophages/foam cells. Three of the samples exhibited lesions consisting of both proliferated SMC and necrotic core with loose connective tissue and foam cells surrounded by fibrous cap. Three samples out of 11 total contained a substantial number of apoptotic cells in the intimal region of the plaque.

**Figure 6:**
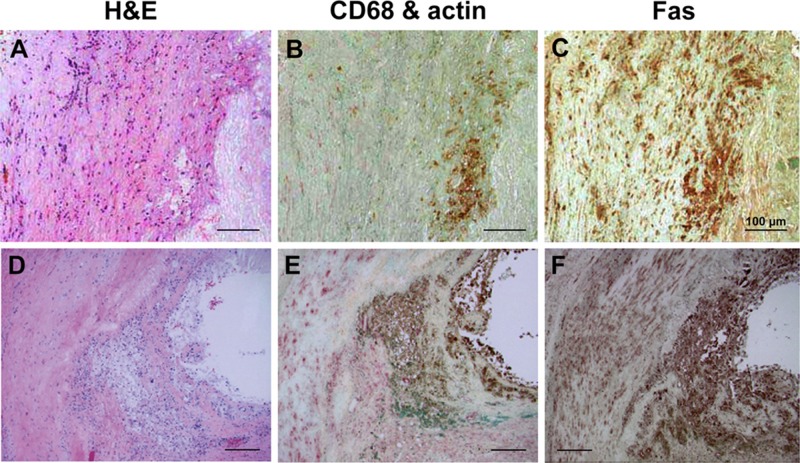
Representative examples of Fas and the presence of inflammatory cells in human aortic fibroatheromatous (type IV) lesions (x40) Two sets of serially sectioned human atherosclerotic tissues with different morphologies (top and bottom panels) are shown. Sections stained with H&E (A, D) as well as with CD68 and a-actin together (B, E) and antibody against Fas (D, F) are shown. CD68 (B, E) and Fas (D, F) are stained brown whereas a-actin is stained red (B, E). In the advanced but less fibrous plaques, inflammatory cells were abundant. The most intense staining for Fas (C, F) can be seen in the CD68-positive foam cell areas (B, E). In the top micrographs, H&E staining (A) shows the acellular necrotic core to be on the right side on all sections whereas the rest of the tissue consists of cells and extracellular matrix representing the fibrous cap. The bottom micrographs show a clear division of loose cells near the lumen most of which are stained positive for CD68 (E). The left side of the lesion contains mainly a-actin-positive smooth muscle cells and extracellular matrix.

We investigated the propensity of plaque cell apoptosis by examining Fas and the plaque cell types in advanced human atherosclerotic tissues that contained the characteristic features of necrotic core as well as foam cells and fibrous caps. Furthermore, we sought to determine if the presence of these molecules was related to lesion morphology. **Figure 6** shows two sets of representative examples of serial sections from two morphologically distinct human atherosclerotic lesions. The tissue sections were stained with H&E (**Figure 6** [A, D]), antibodies against macrophage (**Figure 6**, B and E, brown stain), α-actin (**Figure 6** [B, E], red stain) and Fas (**Figure 6** [C, F]). As a negative control for Fas staining we used an isotype-matched irrelevant primary antibody that showed no staining (data not shown). The neointima contained numerous Fas positive cells (**Figure 6** [C, F]) in both CD68- and a-actin-positive areas of both sets of plaques. However, the most intense staining for Fas was found co-localized with CD68-positive macrophage/foam cells in or around the areas of the plaque that exhibited the characteristic features of necrotic core.

## CONCLUSION

The interactive role of Fas/Fas ligand in atherosclerosis has been studied by many researchers after it was established that both Fas and its receptor Fas ligand are indeed present in human atherosclerotic plaques^8,10^. However, there has been considerable controversy whether the Fas/Fas ligand pathway is pro- or anti-atherogenic. For example, over-expression of Fas ligand in the vasculature can result in acceleration of atherosclerosis^37^ or it can result in decreased neointima formation and atherosclerosis^38,39^. Furthermore, the role of this pathway specifically in leukocytes vis-à-vis atherogenesis has not been explored. Our goal was to examine the role of leukocyte-specific Fas expression in atherosclerosis by using a well-established BMT technique^31,40^ . Detailed morphological analysis during lesion progression revealed that the fibrous area of atheromatous lesion in the mice given lpr bone marrow was appreciably smaller and thinner compared to lesions in the mice given wild-type bone marrow.

A major constituent of the fibrous cap is the extracellular matrix, such as fibrillar collagens, elastin and proteoglycans that are produced primarily by SMCs^41,42^. Inflammatory cytokines such as transforming growth factor-b and interferon-γ produced by leukocytes in the atheroma can regulate the synthesis of these matrix components^43^. Macrophages, being the most abundant immune cell type in the atherosclerotic lesion, probably contributed most to the matrix remodeling that we observed in our experimental mice. Aside from macrophages, however, other candidate immune cells that can cause inflammatory changes in lesions are T, B and NK cells. The lack of Fas is thought to cause a defect in the elimination of self-reactive T cells in their early differentiation in lpr mice^3^. In this regard, these self-reactive T cells that lack both CD4 and CD8 within the thymus express pro-inflammatory cytokines such as interferon-γ and tumor necrosis factor-α that can affect the atherogenic process^44,45^. In the vasculitis model of MRL/lpr mice, it has been postulated that injury to the vessel wall is caused by B-cell-derived immune complexes^14^ (although the injury is seen only in certain genetic backgrounds). It is also known that the cytotoxic activity of NK cells in lpr mice is impaired^46^. Thus, it is possible that these other bone marrow-derived cell types from lpr mice may have contributed to the disease process.

In our study, apoptotic cells were sparsely observed in earlier lesions but became more abundant in more advanced stages of murine atherosclerosis. TUNEL-positive cells were less prominent at later stages of the disease in the lpr-BMT mice. In early lesions (11 weeks on high fat diet) from both lpr-BMT and WT-BMT mice similar numbers of MOMA-2 positive monocyte/macrophages were observed at the luminal surface of the lesion as well as in the subendothelial space (data not shown) indicating that monocyte/macrophage expression of Fas did not influence early monocyte/macrophage attachment to arterial endothelial surface. In addition, our observation that morphological characteristics of fatty streak and intermediate lesions were similar between the lpr-BMT and the WT-BMT mice (data not shown) suggests that leukocyte Fas deficiency had only a minor role in earlier stages of atherosclerotic development. Our observation that the presence of apoptotic cells in atheromatous plaques is relatively scarce is consistent with previous reports of atherosclerosis in humans^34,35^ and mice^36^.

Our examination of the human atheromatous lesion revealed that Fas was expressed throughout the lesion but was particularly concentrated in regions where CD68-positive macrophages were present (**Figure 6**). This indicates that the Fas/Fas ligand pathway is probably important for macrophages due to the inflammatory nature of this cell type. If differentiated and activated lipid-laden macrophages cannot undergo apoptosis, it can be assumed that they will eventually undergo necrosis^47,48^. This will cause the release of numerous growth factors and catabolic enzymes that can potentially change the dynamics of the lesion environment. Necrotic cell death may cause unorganized elastin matrix degeneration and reduced or chaotic matrix rebuilding which may lead to increased necrotic core, decreased fibrous cap formation and ultimately to plaque instability^49^. In contrast, programmed apoptotic cell death may lead to systematic matrix metabolism and successful matrix remodeling that would eventually lead to plaque stability in advanced atherosclerosis. Our findings suggest that macrophage death through Fas in human atherosclerotic lesions may be an important determinant in the organization of the lesion morphology.

It is not clear at this time if the link that we observed between the absence of Fas on immune cells and the morphological changes in lesions is clinically relevant. Because the murine model of atherosclerosis lacks the plaque rupture feature it is difficult to determine if there are any clinical consequences to the changes in lesion morphology caused by the lack of Fas. Nevertheless, the morphological differences that we observed between the lpr-BMT and WT-BMT mice as well as the identification of several morphological features in certain areas of advanced human atheroma suggest Fas expression by the intimal macrophages can indeed affect lesion morphology.

## KEY POINTS

**◊** **Overall atherosclerotic lesion area is not altered by defective Fas expression on immune cells**

**◊** **Defective Fas expression on macrophages causes fibrous areas in atherosclerotic plaque to be smaller**

**◊** **Smaller fibrous area may lead to more vulnerable plaques that are known to be prone to plaque rupture**
